# Enhanced Optical Transmission through a Hybrid Bull’s Eye Structure Integrated with a Silicon Hemisphere

**DOI:** 10.3390/nano13131935

**Published:** 2023-06-25

**Authors:** Yueyang Liu, Jiukai Fang, Yuwen Lin, Shengnan Shi, Chengzhe Di, Shan Zhang, Mingqi Sun, Yanpeng Shi, Yifei Zhang

**Affiliations:** School of Microelectronics, Shandong University, Jinan 250100, China

**Keywords:** extraordinary optical transmission, surface plasmon, terahertz, bull’s eye structure, Mie resonance

## Abstract

In this work, we demonstrate a novel structure that can generate extraordinary optical transmission with a silicon hemisphere placed on a conventional bull’s eye structure. There is a single subwavelength aperture surrounded by concentric periodic grooves on a substrate. The extraordinary optical transmission in this work is realized by the coupling of the surface plasmon polaritons in the periodic grooves and the localized electromagnetic field generated by the Mie resonance in the silicon hemisphere. The maximum normalized-to-area transmission peak can reach up to 662 with a decreasing device area and size. The electromagnetic field distribution at different geometry parameters is analyzed to clarify the mechanisms of the work in this paper. Additionally, the use of dielectric material in the aperture can avoid ohmic losses of metal material compared with the conventional one, which may suggest that a wider range of bull’s-eye-structure applications is possible.

## 1. Introduction

In optical systems, reducing reflectivity is usually an important way of enhancing transmission. Based on optical interference, anti-reflection film is mostly used in conventional transmission enhancement. However, great attention should be focused on the defects of conventional anti-reflection dielectric film, such as the corrosion resistance of thin film materials, the compatibility between the substrate and film layer, and so on. In recent years, as micro- and nano-optics developed, it has been shown that micro- and nano-devices play an important role in transmission improvement due to their optical operability. Through the interaction between devices of micro-nano size and the optical field, the recognition, sensing and exchange of optical information as well as enhanced optical transmission can be realized [1,2].

The interest of enhanced optical transmission (EOT) has been broadly awoken for some time. Since Ebbesen first observed EOT in metal sub-wavelength hole arrays [3], the problem of how to enhance optical transmission through subwavelength holes has been widely researched. Ebbesen’s experiment proved that the EOT phenomenon is due to the coupling of incident light and surface plasmons (SPs). This provides a new method for further applications of novel photon devices such as biochemical sensing [4]. As many studies have verified, EOT can be realized by a subwavelength aperture on a metal film on account of the surface polariton plasmons (SPPs) [5,6]. This plays an important role in the design of subwavelength micro-structured photonic devices [7] and plasmonic imaging lithography [8] because it can locally amplify light energy in the near field.

In addition to the subwavelength-hole array structure, the bull’s eye structure was also proven to realize an enhanced optical transmission. Thio and his group reported EOT through a bull’s eye structure with an aperture on a corrugated metal surface [9]. When incident light resonates with surface plasmons on the corrugated metal surface surrounding an aperture, optical transmission through a subwavelength aperture in a metal film is greatly enhanced. They claimed that the aperture acted as a novel probe, providing a brand new method for optimizing transmission enhancement. Many studies have revealed the mechanism of EOT through the bull’s eye structure. Lezec’s work demonstrated that enhanced transmission is boosted due to the coupling between periodic grooves and SPs [10]. To couple free propagating light with surface plasmons, a periodic structure can be created on the surface to ensure that both energy and momentum are conserved. The high transmission efficiencies of the bull’s eye structure lead it to being applied in high-speed photodetectors [11,12,13], plasmonic focusing [14], molecular sensing [15] and other fields [16]. Introducing particles to subwavelength holes to further enhance optical transmission has been widely studied recently. Wang’s group proposed a novel structure with coupling between a plasmonic nanohole array and a nanorod array. A Ag nanorod was placed in the middle of a Ag nanohole in which the Ag nanorod acted as an antenna. Strong polarization-dependent optical properties and a transmission of 68% were achieved [17]. In 2008, a structure with a square metallic patch centered in a square aperture was investigated by Bao, and they showed the tunable properties of enhanced transmission through ring-shaped apertures [18].

Accordingly, the bull’s eye structure can also be integrated with particles functioning as antennas. Ishihara’s research shows that periodic grooves can be used as an antenna with the bull’s eye structure to concentrate light energy in the central aperture [11], so as to make incident light resonate with electrons on the metal surface and amplify light energy. At present, the bull’s eye structure has been studied in the visible range [19], and many different antennas have been integrated with the periodic optical grating, such as fan-rod antennas [20], bow-tie shaped antennas [19], and metallic nanoantenna rings [21]. A. Degiron’s work proved that the EOT of the bull’s eye structure can be divided into three independent steps [22]: coupling input, transmission of aperture and coupling output. Sun’s work [23] proved that transmission can be enhanced by placing a gold hemisphere on the central hole of the bull’s eye structure on account of the interaction of SPPs generated by the periodic grooves between the LSPRs excited by the gold hemisphere. The maximum normalized-to-area transmission was significantly improved to 556 when the number of grooves reached 20. Compared with the structure without a gold hemisphere, the transmission of the hybrid one can reach the same transmission with fewer grooves. Through the magnetic dipole resonance in the Mie resonance of the silicon particle, Song’s work [24] demonstrated that the two silicon particles placed on both sides of the subwavelength hole array can be seen as magnetic dipole antennas concentrating electro-magnetic energy to enhance transmission. T. Sugaya [25] formed a dielectric material on the aperture of the bull’s eye structure, which confirmed that the transmittance with the dielectric material was greater than the structure without the dielectric material because the SPPs interacted strongly with the dielectric material. What can be concluded from reference [25] is that the dielectric particle can obviously improve the performance of the bull’s eye structure.

In this work, we propose a hybrid bull’s eye structure with a silicon hemisphere placed on the aperture surrounded by concentric periodic grooves. LSPRs excited by metal particles can be coupled with SPPs generated on periodic grooved surfaces to enhance transmission [23], but metallic materials such as gold and silver have non-negligible ohmic losses, which limits their practical application. Gustav Mie’s research [26] found that when electromagnetic waves irradiated dielectric particles, they would interact with each other to achieve an electric or magnetic field response. Compared with metal particles, dielectric particles could generate the resonance of the electric dipole and magnetic dipole at the same time. As proven by Song’s work [24], the silicon resonators can generate Mie resonance and interact with the SPPs on the gold film. Accordingly, in the structure proposed in this paper, silicon hemispheric particles can generate Mie resonance under the irradiation of electromagnetic waves, acting as magnetic dipole antennas to converge electromagnetic energy, and are coupled with SPPs excited by periodic grooves to enhance transmission. Its maximum normalized-to-area transmission can reach up to 662, and the use of silicon particles can avoid the ohmic loss of metal particles, making this structure promising for practical applications of the bull’s eye structure.

## 2. Materials and Methods

**Figure 1:**
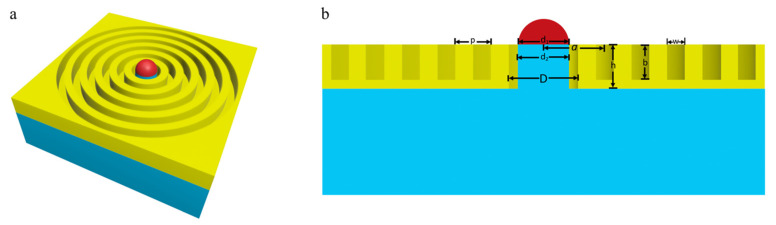
Schematic representation of the proposed structure. (**a**) The 3D schematic of the hybrid bull’s eye structure integrated with a silicon hemisphere (the red one). (**b**) The section in the direction of the XZ view with D=18 μm, thickness of gold film (the yellow one) h=5 μm, support column (the blue one) diameter $d_{2}=16\;\mu m$, width of periodic grooves w =20.5 μm, distance from the center of the first groove to the center of the aperture a =60 μm, depth of grooves b=4 μm, period of grooves p and diameter of the hemisphere d_1_.

The structure of the bull’s eye antenna we proposed is shown in Figure 1. The structure consists of a silica substrate, silicon hemisphere, central pore, gold film and periodic grooves. The SiO_2_ substrate is covered with a layer of gold film (the thickness is fixed at 5 µm), and a central hole and several periodic grooves are on the gold film. The silicon hemisphere is supported by a SiO_2_ pillar inside the central hole. The periodic grooves and the central hole have the same center. The center hole diameter is D, the hemisphere diameter is d1, and the strut diameter and height are d2 and h. The period of the groove, the width of the groove, the depth of the groove, the number of grooves and the distance from the center of the first groove to the center of the circle are described as p, w, b, N, and a [23]. The three-dimensional finite-difference-time-domain (FDTD) method is numerically used to calculate the transmission enhancement spectra and field distribution. A plane wave polarized along the x direction illuminates the whole device with a wavelength ranging from 43 to 75 µm. The perfectly matched layer (PML) boundary condition is used in the x, y, and z directions. An auto-shutoff minimum of 1 × 10^−5^ in the simulations is adopted to trade between accuracy, RAM capacity, and running time. The low-loss dielectric permittivity of the SiO_2_ pillar and substrate is 1.5. The optical constants of gold are characterized by the Drude mode [27], and the low-loss silicon permittivity is described by the Palik model. There is a spatial step discretization around the central hole of 0.8 × 0.8 × 0.4 µm^3^ and a spatial step discretization around the grooves of 6 × 6 × 1 µm^3^. The transmission enhancement is normalized to the area of the central hole. The thermal evaporation technique and focused ion beam (FIB) can be used to fabricate the proposed structure. First, FIB can be used to etch the SiO_2_ substrate to form a pillar as the column, after which a layer of gold film can be deposited on the substrate using thermal evaporation technique, followed by etching the central hole and grooves with FIB [28]. Finally, the silicon hemisphere can be connected to the pillar through a bonding process [23].

## 3. Results and Discussion

**Figure 2:**
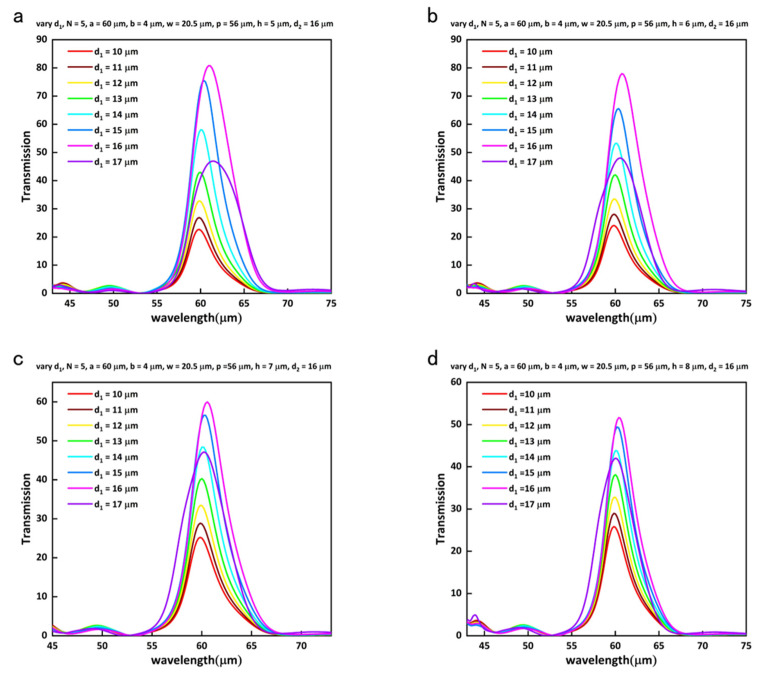
Variation of transmission characteristics with the change of h and d_1_. (**a**) The variation transmission characteristics with the change of d_1_ at h = 5 µm. (**b**) The variation transmission characteristics with the change of d_1_ at h = 6 µm. (**c**) The variation transmission characteristics with the change of d_1_ at h = 7 µm. (**d**) The variation transmission characteristics with the change of d_1_ at h = 8 µm.

**Figure 3:**
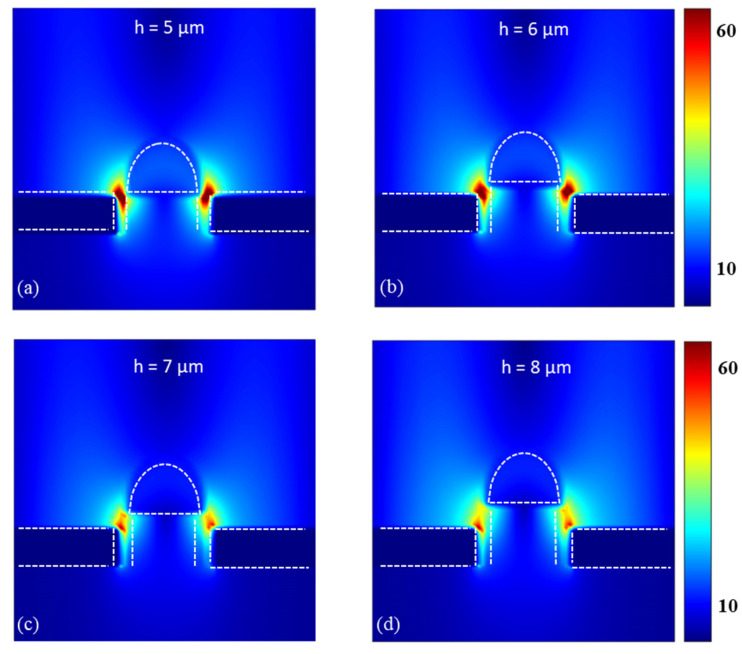
The XZ cross-sectional electric field distributions when d_1_ = 16 µm under the change of h, for excitation at λ = 62 µm. The dotted lines indicate the position of the gold film, column, and hemisphere. (**a**) The XZ cross-sectional electric field distributions when d_1_ = 16 µm and h = 5 µm. (**b**) The XZ cross-sectional electric field distributions when d_1_ = 16 µm and h = 6 µm. (**c**) The XZ cross-sectional electric field distributions when d_1_ = 16 µm and h = 7 µm. (**d**) The XZ cross-sectional electric field distributions when d_1_ = 16 µm and h = 8 µm.

Figure 2 illustrates the normalized-to-area transmission characteristics of the hybrid bull’s eye structure as a function of the dimensions h and d_1_. Specifically, Figure 2a displays the enhancement in transmission as d_1_ varies from 10 µm to 17 µm, while keeping the number of grooves fixed at 5 and the height of the support column at 5 µm. The transmission enhancement can be obtained at the same peak position by altering the size of the height of the support column. It can be clearly seen from Figure 2 that similar transmission curves are obtained at different heights, which means that the heights of the support column in the hole only affect the maximum transmission. This is because there is a certain area of the aperture that can be used for optical transmission when the diameter of the hemisphere is fixed. When the height of the column increases, it is obvious that the maximum transmission decreases. The transmission peak at h = 8 µm and d_1_ = 16 µm is about 63% of the transmission peak at h = 5 µm and d_1_ = 16 µm. The normalized electric field intensity ${\left|E\right|}^{2}$ [20] at h = 5 µm and d_1_ = 16 µm is enhanced 2.65 times by h = 8 µm and d_1_ = 16 µm, which leads to a 1.72-fold enhancement in transmission. The reason for this is that the distance between the hemisphere and top sharp corner of the hole increases, so that the interaction between the Mie resonance of the hemisphere and the SPPs of the concentric periodic grooves has been weakened, as can be seen from Figure 3, which shows that the maximum values of the electric field in the gap improved as the height decreases. Meanwhile, there is an obvious red shift in the transmission curves when d_1_ increases. The secondary radiation is enhanced, causing the electrons to lose energy through a damping effect. This results in a broadening and red shift of the peak [29].

To better reveal the mechanism of the calculated transmission spectrum, the electric field distribution at different heights is plotted in Figure 3. A strong resonance is observed in the gap between the hemisphere and the aperture. This implies that the Mie resonance of the silicon particle can interact with the SPPs of gold film, which leads to optical transmission enhancement. When the height is fixed, the transmission can be enhanced as the diameter of the hemisphere increases. However, the transmission begins to decrease as the hemisphere diameter exceeds the strut diameter by 16 µm. When $d_{1}<17\;\mu m$, the central hole is not occluded by the hemisphere, so the area light passing through would be fixed at 17π µm^3^. When h = 5 µm, the maximum transmission at d_1_ = 16 µm is around 80, which is 3.6 times higher than the maximum transmission at d_1_ = 10 µm; this is because a larger hemisphere has a higher transmission, since there are more electrons in larger hemispheres to produce stronger Mie resonance and since the edge of the hemisphere is closer to the periodic grooves and more strongly coupled. When d_1_ is 17 µm (larger than the diameter of the column), the center hole is partially blocked by the hemisphere, making the area for light to pass through decrease to 7.75π µm^3^, resulting in a decrease of 1.7 times (when h = 5 µm) in transmission enhancement compared with d_1_ = 16 µm. Moreover, the maximum transmission with a d_1_ of 17 µm is larger compared with a d_1_ of 13 µm and below, from which we can conclude that it is the volume of the hemisphere that plays a dominant role in THz transmission enhancement when $d_{1}\leq 13\;\mu m$, while the distance between the hemisphere and top sharp corner of the hole dominates when $13\;\mu m<d_{1}<17\;\mu m$. As for d_1_ = 17 µm, the area of the aperture that can be used for optical transmission would be the key factor.

**Figure 4:**
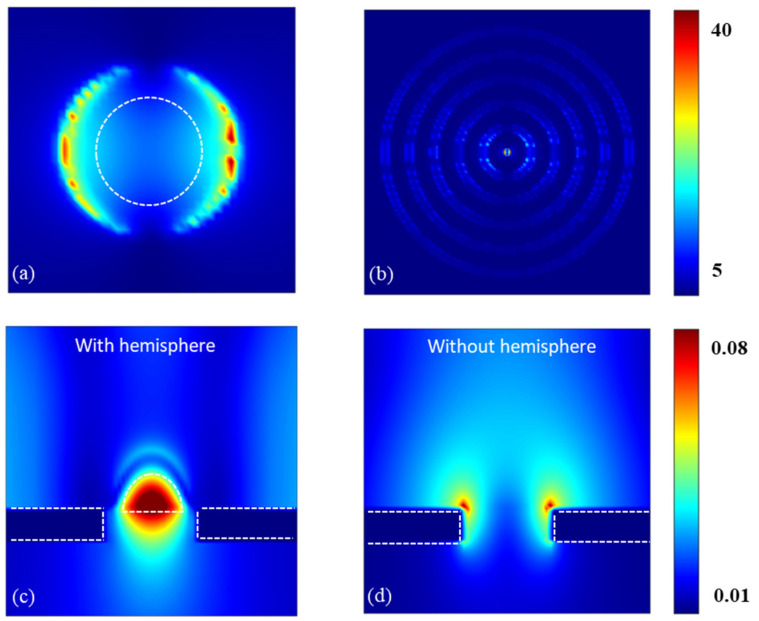
Plot of instantaneous electric and magnetic field distribution for excitation at λ = 62 µm. (**a**) The XY view of the electric field distribution near the aperture. The dotted line indicates the position of the hemisphere. (**b**) The XY view of the electric field distribution on the surface of the gold film. (**c**) The XZ view of the magnetic field distribution with silicon hemisphere. The dotted lines indicate the position of the hemisphere and gold film. (**d**) The XZ view of the magnetic field distribution without silicon hemisphere. The dotted line indicates the position of the gold film.

In order to further study the influence of hemispheres and grooves on transmission enhancement, the XY view of the electric field distribution and the XZ view of the magnetic field distribution with/without a silicon hemisphere are plotted in Figure 4. As shown in Figure 4a, one can see that most of the resonance is concentrated in the edge of the hemisphere, indicating that the hemisphere can couple the incident light with surface plasmons. Figure 4b shows that the electric field is highly confined in the grooves and the central hole. The normalized electric field intensity ${\left|E\right|}^{2}$ [20] in the grooves is enhanced around 31.7 times by the area between the grooves, which reveals the focusing of the SPPs in the bull’s eye structure by the periodic grooves. What can be concluded from this is that the grooves can act as antennas [30] to harvest light and propagate it to the central aperture, coupling the SPPs with the Mie resonance of the silicon hemisphere. This process can lead to more light being concentrated in the aperture to be coupled, so that a transmission enhancement can be achieved. As shown in Figure 4c, a strong magnetic field can be observed in and under the hemisphere, which shows that the energy can be significantly coupled through the hole and transmitted out. Compared with Figure 4c, Figure 4d illustrates that the energy can only be concentrated at the edge of the gold film without the silicon hemisphere. With it being harder for the energy to propagate through the aperture, the transmission peak without the hemisphere is much smaller than that with the hemisphere. It is worth mentioning that the hemisphere plays a significant role in the improvement of transmission enhancement through the comparison shown in Figure 4c,d.

**Figure 5:**
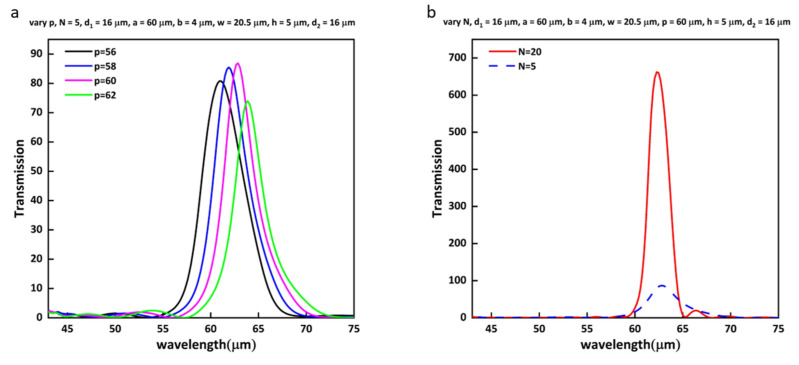
(**a**) Effect of the period of grooves on the transmission enhancement spectra. (**b**) Effect of the number of grooves on the transmission enhancement spectra.

Figure 5a shows the transmission enhancement spectra at different periods for five grooves. The transmission peak wavelength $\lambda_{\mathrm{spp}}$ is introduced by equation $\lambda_{\mathrm{spp}}=p\sqrt{\frac{\varepsilon_{m}\varepsilon_{d}}{\varepsilon_{m}\;+\;\varepsilon_{d}}}$, where p represents the period of the grooves. $\varepsilon_{m}$ and $\varepsilon_{d}$, respectively, represent the relative dielectric constant of Au and air [31]. It can be seen from Figure 5a that the transmission peak red-shifts as p increases. The reason for this is that the periodic grooves can couple the incident wave with the SPPs and transmit it at a wavelength close to the period of the grooves [32]. As shown in the spectra, the transmission enhancement reached the maximum at the period of 60 µm. We can also conclude from the figure that p is smaller than the resonance wavelength, which does not correspond to the Bragg coupling condition because the penetration of the field in the grooves can increase the optical path [33]. Figure 5b shows the EOT curves under N = 5 and N = 20. It is obvious that the maximum transmission enhancement at N = 20 can reach 662, which is 7.7 times higher than that at N = 5. What accounts for this is that an increasing number of grooves can collect an extending range of incident light and squeeze it into the central aperture.

## 4. Conclusions

In this work, we propose a hybrid bull’s eye structure with a silicon hemisphere placed on an aperture surrounded by concentric periodic grooves. Through the interaction between the silicon hemisphere and the gold grooves, the normalized-to-area transmission has been significantly enhanced. Introducing a silicon hemisphere to the conventional bull’s eye structure can avoid a non-negligible ohmic loss of metallic materials. Silicon hemispheric particles that generate Mie resonance under electromagnetic wave irradiation can act as magnetic dipole antennas to converge electromagnetic energy. EOT can be realized by the coupling between the Mie resonance of the silicon hemisphere and SPPs excited by the periodic grooves. The maximum normalized-to-area transmission when the number of grooves is 20 can reach 662, which is seven times as great as when N = 5. The use of silicon particles can avoid the ohmic loss of metal particles and promote the practical application of the bull’s eye structure.

## Data Availability

Not applicable.
